# Genetic analysis of osteogenesis imperfecta in the Palestinian population: molecular screening of 49 affected families

**DOI:** 10.1002/mgg3.331

**Published:** 2017-11-18

**Authors:** Osama Essawi, Sofie Symoens, Maha Fannana, Mohammad Darwish, Mohammad Farraj, Andy Willaert, Tamer Essawi, Bert Callewaert, Anne De Paepe, Fransiska Malfait, Paul J. Coucke

**Affiliations:** ^1^ Department Master Program in Clinical Laboratory Science Birzeit University Birzeit Palestine; ^2^ Center for Medical Genetics Ghent University Ghent Belgium; ^3^ Dr. Al Rantisi Specialized Children Hospital Gaza Palestine; ^4^ Rafidia Surgical Hospital Nablus Palestine

**Keywords:** Autosomal dominant, autosomal recessive, next‐generation sequencing, osteogenesis imperfecta

## Abstract

**Background:**

Osteogenesis imperfecta (OI) is a heterogeneous hereditary connective tissue disorder clinically hallmarked by increased susceptibility to bone fractures.

**Methods:**

We analyzed a cohort of 77 diagnosed OI patients from 49 unrelated Palestinian families. Next‐generation sequencing technology was used to screen a panel of known OI genes.

**Results:**

In 41 probands, we identified 28 different disease‐causing variants of 9 different known OI genes. Eleven of the variants are novel. Ten of the 28 variants are located in *COL1A1*, five in *COL1A2*, three in *BMP1*, three in *FKBP10*, two in *TMEM38B*, two in *P3H1*, and one each in *CRTAP*,*SERPINF1*, and *SERPINH1*. The absence of disease‐causing variants in the remaining eight probands suggests further genetic heterogeneity in OI. In general, most OI patients (90%) harbor mainly variants in type I collagen resulting in an autosomal dominant inheritance pattern. However, in our cohort almost 61% (25/41) were affected with autosomal recessive OI. Moreover, we document a 21‐kb genomic deletion in the *TMEM38B* gene identified in 29% (12/41) of the tested probands, making it the most frequent OI‐causing variant in the Palestinian population.

**Conclusion:**

This is the first genetic screening of an OI cohort from the Palestinian population. Our data are important for genetic counseling of OI patients and families in highly consanguineous populations.

## Introduction

Osteogenesis imperfecta (OI) or brittle bone disease is a rare heterogeneous hereditary disorder with an incidence of 1:15,000 to 1:25,000 births (Stoll et al. 1989; Martin and Shapiro 2007). The clinical hallmark of OI is a low bone mass that causes bone fragility, easy fracturing, and growth impairment. Other features may include blue sclerae, dentinogenesis imperfecta, and hearing loss (van Dijk and Sillence 2014). The clinical heterogeneity of OI ranges from hardly detectable mild OI with few fractures to perinatal lethality. Autosomal dominant (AD), autosomal recessive (AR), and X‐linked inheritance patterns have been described previously. The current clinical classifications elaborating on the OI classification of 2010 reveal the importance of phenotyping for classifying and diagnosing OI (Warman et al. 2011; van Dijk and Sillence 2014; Bonafe et al. 2015). Forlino and Marini (2016) subdivide OI genes in five functional groups according to the pathway and mechanism in which they are involved. We present our results according to these functional groups. However, several reported OI genes cannot be classified, including *TAPT1*,* SEC24D*,* P4HB*,* SPARC*, and *MBTPS2* (OMIM#300294) (Garbes et al. 2015; Mendoza‐Londono et al. 2015; Rauch et al. 2015; Symoens et al. 2015; Lindert et al. 2016).

Middle Eastern populations, especially Arabs, are highly consanguineous because of cultural reasons. Population‐based surveys show consanguinity rates of 20–50% in all marriages in Arab countries (Tadmouri et al. 2009). In Palestine, this rate has been estimated to be about 40% (Assaf and Khawaja 2009; Tadmouri et al. 2009; Sirdah 2014). Consequently, AR disorders are common in these populations. In contrast to the high frequency of the AD forms caused by defects in the structure or quantity of type I collagen in nonconsanguineous populations (Byers and Pyott 2012; Rohrbach and Giunta 2012), AR forms of OI are expected to be more common in highly consanguineous populations. The many AR genes identified in consanguineous families have revealed new pathogenic mechanisms. Some genes have been subjected to intensive study, such as *CRTAP*,* P3H1*, and *PPIB*, which encode components of the collagen prolyl 3‐hydroxylation complex (Marini and Blissett 2013; Homan et al. 2014; Forlino and Marini 2016) and *BMP1*, which encodes the C‐propeptidase of type I procollagen and causes AR OI through a procollagen processing defect. In addition, BMP1 activates lysl oxidase, which has a critical role in collagen cross‐linking (Panchenko et al. 1996; Borel et al. 2001). Other genes have not been fully explored: (i) *SERPINF1*, encoding the pigment epithelium‐derived factor protein (PEDF), which has a crucial role in bone homeostasis and osteoid mineralization (Minillo et al. 2014); (ii) *SP7*, encoding the transcription factor Sp7 protein; (iii) *WNT1*, encoding the proto‐oncogene Wnt‐1 protein; and (iv) *CREB3L1*, encoding the endoplasmic reticulum stress transducer “old astrocyte specifically induced substance” (OASIS) (Lapunzina et al. 2010; Keupp et al. 2013; Symoens et al. 2013). A recently identified AR OI gene is *TMEM38B*, encoding the TRIC‐B protein (Shaheen et al. 2012). It has been proposed that the TRIC‐B channel acts as a counter ion to facilitate the Ca^2+^ efflux from the endoplasmic reticulum (ER) mediated by inositol 1,4,5‐trisphosphate receptors (IP3Rs) (Fink and Veigel 1996). Impaired bone mineralization and insufficient collagen matrix in bones have been reported in TRIC‐B knockout mice, which die immediately after birth from respiratory complications. Moreover, it was proposed in the same study that TRIC‐B knockout osteoblasts inhibit IP3R‐mediated Ca^2+^ release, leading to impaired Ca^2+^ signaling and Ca^2+^ store overload (Zhao et al. 2016). A recent study based on data obtained from cells of OI patients reported higher ER stress accompanied by defective matrix collagen due to the decreased synthesis, secretion, and deposition of type I collagen, in addition to the impaired assembly and lysyl hydroxylation of procollagen fibers (Cabral et al. 2016). Here, we report for the first time an in‐depth molecular screening of a large cohort of Palestinian OI families and describe a wide range of mostly autosomal recessive variants, with phenotypes ranging from mild to severe.

## Materials and Methods

### Ethical compliance

The study was approved by the ethics committee of Ghent University Hospital (Belgium) and the ethics committee of Birzeit University (Palestine).

### Patients

Forty‐nine Palestinian families with 77 affected family members participated in the study. Participating families were distributed all over Palestine, with 38 residing in the West Bank and 11 in Gaza. Thirty‐two (65%) of the 49 families were consanguineous. Blood samples were obtained from affected individuals after obtaining appropriate informed consent from the participant and/or the legal guardians.

### Cell culture and isolation of DNA and RNA

Genomic DNA was isolated and purified from whole EDTA blood by Qiagen DNeasy Kit using standard protocols (Qiagen, Frankfurt, Germany). Skin biopsies were obtained from probands affected by a disease‐causing splice variant in *FKBP10*. RNA was isolated with the RNeasy mini kit (Qiagen). Subsequently, cDNA was synthesized using the M‐MLV cDNA synthesis kit according to the manufacturer's instructions (Qiagen).

### Analysis strategies

A total of 82 primer sets were developed to amplify the exons and their intron boundaries of OI panel 1, associated with AD OI, including *COL1A1* (OMIM# 120150), *COL1A2* (OMIM# 120160), and *IFITM5* (OMIM# 614757); 185 primer sets were developed to amplify OI panel 2, associated with AR OI, including *SERPINF1* (OMIM# 172860), *SERPINH1* (OMIM# 600943), *P3H1* (OMIM# 610339), *FKBP10* (OMIM# 607063), *TMEM38B* (OMIM# 611236), *CRTAP* (OMIM# 605497), *SP7* (OMIM# 606633), *BMP1* (OMIM# 112264), *CREB3L1* (OMIM# 616215), *PLOD2* (OMIM# 601865), *TAPT1* (OMIM# 612758), *PPIB* (OMIM# 123841), *SEC24D* (OMIM# 607186), *P4HB* (OMIM# 176790), and *SPARC* (OMIM# 182120). All primer sequences were obtained from Pxlence (Dendermonde, Belgium). The coding regions and flanking introns were amplified using a 2720 Thermal Cycler (Applied Biosystems, Inc., Foster city, CA, USA). Depending on the clinical presentation and family history, either OI panel 1 or OI panel 2 was analyzed. If the screening was negative, the other panel was investigated. Samples (50 ng DNA) were prepared using the Nextera sample preparation protocol (Nextera XT DNA Sample Prep Kit) (Illumina, Inc., San Diego, CA, USA) and sequenced on a MiSeq instrument (Illumina, Inc.). Alterations were confirmed by bidirectional Sanger sequencing using an ABI3730XL sequencer (Applied Biosystems, Inc.). Nomenclature is based on the HGMD guidelines and refers to NCBI reference sequence NM_000088.3/NP_000079.2 for *COL1A1*, NM_000089.3/NP_000080.2 for *COL1A2*, NM_006129.4/NP_006120.1 for *BMP1*, NM_006371.4/NP_006362.1 for *CRTAP*, NM_022356.3/NP_071751.3 for *P3H1*, NM_018112.1/NP_060582.1 for *TMEM38B*, NM_001207014.1/NP_001193943.1 for *SERPINH1*, NM_021939.3/NP_068758.3 for *FKBP10*, and NM_002615.5/NP_002606.3 for *SERPINF1*. Pathogenic variants were evaluated with the Alamut software (Alamut Visual, Interactive Biosoftware, Rouen, France) and the Mutalyzer software (https://mutalyzer.nl/batchNameChecker). The results were submitted to the OI variant database (https://oi.gene.le.ac.uk) (Dalgleish 1997, 1998).

To identify disease‐causing variants, all alterations were filtered against the OI variant database (https://oi.gene.le.ac.uk), the dbSNP database (Sherry et al. 2001), and the ExAc database (Lek et al. 2016) (MAF <1%). Variants were considered pathogenic if they satisfied previously published criteria (Symoens et al. 2012).

### Linkage analysis

Microsatellite markers within the ±1 Mb flanking the gene being investigated were selected from the Genethon and Marshfield genetic map for linkage analysis. Markers and primer sequences are shown in Table S1. PCR reactions were performed (reaction conditions are available upon request). One μL PCR product was added to 10 μL of a mixture of GeneScan 500 LIZ Size Standards (Applied Biosystems, Inc.) and formamide, and analyzed on an ABI3730XL Genetic Analyzer (Applied Biosystems, Inc.). The results were analyzed using GeneMapper software V5.0 (Applied Biosystems, Inc.).

## Results

### Alterations causing defects in collagen synthesis, structure, or processing

We identified variants in *COL1A1*,* COL1A2*, or *BMP1* in 19 of the 49 Palestinian OI probands (Table 1). Eleven of 19 probands harbored 10 different *COL1A1* disease‐causing variants. Two probands had glycine substitutions in the *α*‐helical region c.3226G>A p.(Gly1076Ser) and c.3118G>A p.(Gly1040Ser), and a third proband carried an aspartate substitution in the C‐propeptide domain c.4237G>A p.(Asp1413Asn). The phenotype of these probands is in agreement with the reports that associate these three substitutions with severe OI (Marini et al. 2007; Bodian et al. 2009; Pyott et al. 2011; Barkova et al. 2015; Lindahl et al. 2015). Three probands had a splice site variant, including variants c.1200+1G>A and c.1299+1G>A, previously reported to cause an in‐frame skip of exons 18 and 19, respectively, and a novel splice site variant, c.3531+1G>T, predicted to cause an in‐frame skip of exon 47. Those three splice site variants were associated with mild to moderate phenotypic features characterized primarily by short stature and recurrent fractures, which is in agreement with previous descriptions (Willing et al. 1993; Benusiene and Kucinskas 2003). Finally, we found four premature termination variants associated with mild phenotypic features. The c.2426dup p.(Ala811Cysfs*10) and c.3749del p.(Gly1250Alafs*81) variants are novel, whereas the c.3567del p.(Gly1190Valfs*49) and c.189C>A p.(Cys63*) variants have been reported previously (Dalgleish 1997, 1998; Lindahl et al. 2015).

**Table 1:** Clinical data overview

Patient ID	Gene	c.DNA variant	Predicted protein	Consanguinity	Sex	Age	Number in pedigree	Recurring fractures	Blue sclera	Hearing loss	Short stature	S & D extremities	Pectus deformity	Kyphoscoliosis	Contractures	Hernia	Dentinogenesis imperfecta	Wheelchair
AN_001998	*COL1A1*	c.3531+1G>T^a^		–	M	5		15	+	–	+	–	–	–	–	–	–	–
AN_001999	*COL1A1*	c.3226G>A	p.(Gly1076Ser)	–	M	7		>20	+	–	+	+	–	+	–	+	–	+
AN_002000	*COL1A1*	c.3567del	p.(Gly1190Valfs*49)	–	F	3		4	+	–	–	–	–	–	–	–	–	–
AN_005801	*COL1A1*	c.2426dup^a^	p.(Ala811Cysfs*10)	–	F	15		15	+	–	+	+	–	–	–	–	–	–
AN_005802	*COL1A1*	c.4237G>A	p.(Asp1413Asn)	–	M	4		>25	+	–	+	+	+	+	–	–	–	+
AN_005803	*COL1A1*	c.3118G>A	p.(Gly1040Ser)	–	F	7		>20	+	–	+	NA	+	–	+	+	+	+
AN_005804	*COL1A1*	c.1200+1G>A		–	F	2		4	+	–	+	–	–	–	–	–	–	NR
AN_005805	*COL1A1*	c.3749del^a^	p.(Gly1250Alafs*81)	–	M	6	II:1	14	+	+	–	–	–	–	–	–	+	–
					F	4	II:2	6	+	–	–	–	–	–	–	–	–	–
AN_005806	*COL1A1*	c.3567del	p.(Gly1190Valfs*49)	+	M	10	II:3	8	+	–	–	–	–	–	–	–	+	–
AN_005807	*COL1A1*	c.189C>A	p.(Cys63*)	–	M	16		10	+	–	–	–	–	–	–	–	NA	–
AN_005808	*COL1A1*	c.1299+1G>A		–	F	10		>40	+	–	–	–	+	–	–	–	–	+
AN_005809	*COL1A2*	c.1072G>A	p.(Gly358Ser)	–	F	1		5	+	NA	+	+	–	–	+	–	NA	NR
AN_005810	*COL1A2*	c.1031_1033del	p.(Val345del)	–	F	30		>80	+	–	+	+	+	+	–	–	+	+
AN_005811	*COL1A2*	c.3305G>A	p.(Gly1102Asp)	+	F	4		10	+	–	–	+	–	–	–	–	–	–
AN_005812	*COL1A2*	c.1991G>T^a^	p.(Gly664Val)	–	M	40	II:2	>30	NA	–	+	+	–	+	–	–	+	+
					F	14	III:2	>20	+	–	+	+	–	–	–	–	+	+
					F	12	III:3	>15	+	–	+	+	–	–	–	–	+	+
AN_005813	*COL1A2*	c.3034G>A	p.(Gly1012Ser)	–	F	8		18	+	–	+	+	+	–	–	–	+	
AN_005814	*BMP1*	c.688C>G^a^	p.(Arg230Gly)	+	M	23		>25	–	–	+	–	+	+	–	–	–	+
AN_005815	*BMP1*	c.691G>T^a^	p.(Asp231Tyr)	+	M	19	IV:6	>10	–	–	–	–	–	–	–	–	–	–
					M	26	IV:4	6	–	–	–	–	–	–	–	–	–	–
AN_005816	*BMP1*	c.818C>T^a^	p.(Ala273Val)	+	F	10		>100	+	–	+	+	+	+	–	–	+	+
AN_005817	*CRTAP*	c.976C>T^a^	p.(Gln326*)	+	M	9		>40	+	–	+	+	+	+	NA	+	–	+
AN_005818	*CRTAP*	c.976C>T^a^	p.(Gln326*)	+	M	7		>30	+	–	+	+	+	+	NA	+	+	+
0000968	*P3H1*	c.2041C>T	p.(Arg681*)	+	M	8	IV:1	>40	+	–	+	+	+	+	NA	+	+	+
AN_005819	*P3H1*	c.1080+1G>T		+	F	33	II:6	>100	–	+	+	+	+	NA	NA	–	–	+
					F	22	II:10	>100	–	–	+	+	+	+	NA	–	–	+
AN_005820	*TMEM38B*	c.455‐542del	p.(Gly152Alafs*5)	+	M	14	II:4	>20	–	–	–	–	–	–	–	–	–	+
					F	12	II:3	>20	–	–	–	–	–	–	–	–	–	+
					F	15	II:5	3	–	–	–	–	–	–	–	–	–	–
AN_005821	*TMEM38B*	c.455‐542del	p.(Gly152Alafs*5)	+	M	10	V:9	6	–	–	–	–	–	–	–	–	–	–
					F	19	V:6	4	–	–	–	–	–	–	–	–	–	–
					M	20	V:12	9	–	–	–	–	–	–	–	–	–	–
					M	17	V:2	>10	–	–	–	–	–	–	–	–	–	–
AN_005822	*TMEM38B*	c.455‐542del	p.(Gly152Alafs*5)	+	F	1		NA	–	–	–	+	–	–	–	–	–	NR
AN_005823	*TMEM38B*	c.455‐542del\c.507G>A	p.(Gly152Alafs*5)\p.(Trp169*)	–	M	6		15	–	–	–	–	–	–	–	–	+	–
AN_005824	*TMEM38B*	c.455‐542del	p.(Gly152Alafs*5)	+	M	4		5	+	–	–	–	–	–	–	–	–	–
AN_005825	*TMEM38B*	c.455‐542del	p.(Gly152Alafs*5)	+	M	8		6	+	–	–	–	–	–	–	–	+	–
AN_005826	*TMEM38B*	c.455‐542del	p.(Gly152Alafs*5)	+	M	27	II:4	>10	–	–	–	–	–	–	–	–	+	–
AN_005827	*TMEM38B*	c.455‐542del	p.(Gly152Alafs*5)	+	M	7	II:10	4	+	–	–	–	–	–	–	–	+	–
					F	23	II:6	7	–	–	–	–	–	–	–	–	–	–
AN_005828	*TMEM38B*	c.455‐542del	p.(Gly152Alafs*5)	+	M	39	II:2	>10	–	–	–	–	–	–	–	–	+	+
					F	28	II:5	>10	–	–	–	–	–	–	–	–	+	+
AN_005829	*TMEM38B*	c.455‐542del	p.(Gly152Alafs*5)	+	F	4		3	+	–	–	–	–	–	–	–	+	–
AN_005830	*TMEM38B*	c.455‐542del	p.(Gly152Alafs*5)	+	F	3		6	+	–	–	–	–	–	–	–	+	–
AN_005831	*TMEM38B*	c.455‐542del	p.(Gly152Alafs*5)	+	F	7	IV:3	>20	+	–	–	–	–	–	–	–	+	+
					M	13	IV:6	15	+	–	–	–	–	–	–	–	+	+
					F	10	IV:7	5	+	–	–	–	–	–	–	–	–	–
AN_005832	*SERPINH1*	c.314_325del^a^	p.(Glu105_His108del)	+	M	2		4	+	–	NA	NA	+	–	–	–	+	+
AN_005833	*FKBP10*	c.391+4A>T^a^		+	M	41	V:6	>50	+	–	+	+	+	–	+	+	+	+
					F	43	V:5	>50	+	–	+	+	+	–	+	+	+	+
					M	32	V:8	>100	+	–	+	–	+	+	+	–	–	+
					F	30	V:9	>100	+	–	+	–	+	+	+	–	–	+
					F	15	V:12	20	+	–	–	–	+	+	+	–	–	+
					M	14	V:13	15	+	–	–	–	+	+	+	–	+	+
					M	6	V:14	6	+	–	+	+	+	+	+	–	–	+
					F	9	V:3	>12	+	–	–	+	+	+	+	–	–	+
AN_005834	*FKBP10*	c.391+4A>T^a^		+	F	7		>30	–	–	+	–	+	–	+	–	–	+
AN_005835	*FKBP10*	c.1330C>T	p.(Gln444*)	+	F	16	IV:5	>30	+	–	+	+	+	+	+	–	+	+
					F	11	IV:7	>20	+	–	+	+	+	+	+	–	–	+
					M	14	IV:11	>30	+	–	+	+	+	+	+	–	–	+
					M	10	IV:12	>20	+	–	+	+	–	+	+	–	–	+
AN_005836	*FKBP10*	c.1276del^a^	p.(Gln426Argfs*10)	+	M	36	V:2	>15	NA	–	+	+	+	–	+	–	–	+
AN_005837	*SERPINF1*	c.242C>G	p.(Ser81Cys)	+	M	7		>20	–	–	–	–	–	–	–	–	+	–

Nomenclature refers to NCBI RefSeq NM_000088.3/NP_000079.2 for *COL1A1*, NM_000089.3/NP_000080.2 for *COL1A2*, NM_006129.4/NP_006120.1 for *BMP1*, NM_006371.4/NP_006362.1 for *CRTAP*, NM_022356.3/NP_071751.3 for *P3H1*, NM_018112.1/NP_060582.1 for *TMEM38B*, NM_001207014.1/NP_001193943.1 for *SERPINH1*, NM_021939.3/NP_068758.3 for *FKBP10*, and NM_002615.5/NP_002606.3 for *SERPINF1*. Patient ID corresponds to the identifiers found in the OI Variant Database (https://oi.gene.le.ac.uk/). S & D extremities, short and deformed extremities; NA, not available; NR, not relevant.

a Novel mutation.

**Figure 1:**
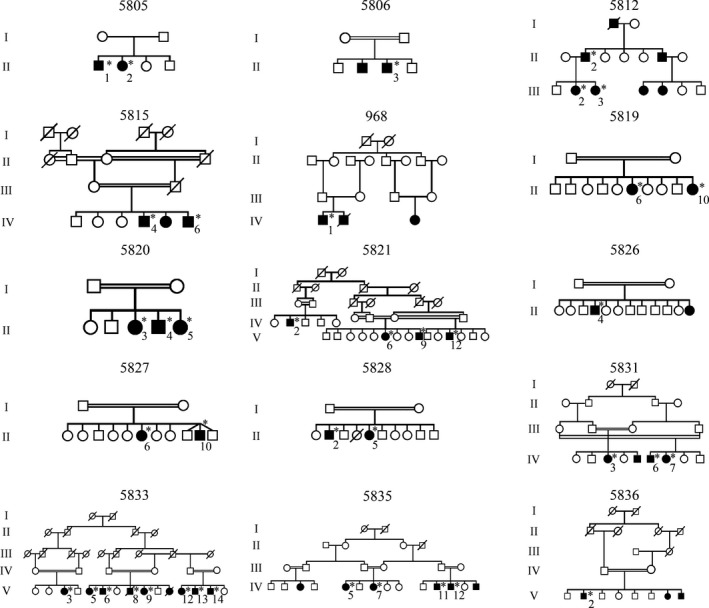
Pedigrees of the families that participated in this study and have more than one OI patient. All relevant family members are indicated. Asterisk (*) indicates patients from whom DNA was obtained.

Five probands had a *COL1A2* variant. Four were glycine substitutions located in the *α*‐helical region of the *α*2(I) chain. One of them, c.1991G>T p.(Gly664Val), was novel, and segregation was confirmed in two affected family members (Fig. 1, 5812). The fifth variant is an in‐frame deletion c.1031_1033del p.(Val345del) causing a severe form of OI. Remarkably, only three probands harboring variants in one of the collagen genes have a positive family history of OI. Mosaicism is suspected in two of those families because there were patients whose parents did not exhibit any OI symptoms (Fig. 1, 5805 and 5806).

Three probands carried novel homozygous missense variants in *BMP1*. The proband with the homozygous variant c.688C>G p.(Arg230Gly) had a moderate to severe phenotype and various deformities necessitating dependency on a wheel chair. The second proband and his affected brother harbored a homozygous missense variant c.691G>T p.(Asp231Tyr) and had a milder phenotype with few fractures and mild deformities (Fig. 1, 5815). The third proband with a homozygous missense variant, c.818C>T p.(Ala273Val), had the most severe phenotype with more than 100 fractures and severe mobility‐limiting deformities.

### Alterations causing defects in collagen modification

A novel homozygous nonsense variant, c.976C>T p.(Gln326*), located in exon 5 of the *CRTAP* gene, was identified in two probands residing in the same geographical region. This variant resulted in a very severe phenotype, including bone and pectus deformities, multiple recurrent fractures, short stature, and congenital hernia.

Two previously reported homozygous variants were detected in the *P3H1* gene, one nonsense variant, c.2041C>T p.(Arg681*) (Pepin et al. 2013), and a splice site variant, c.1080+1G>T (Fig. 1, 968 and 5819). Both variants resulted in a severe OI phenotype, with extremely short stature, short and deformed extremities, and significant mobility impairment (Table 1). Moreover, a family history of neonatal and childhood death was reported.

**Figure 2:**
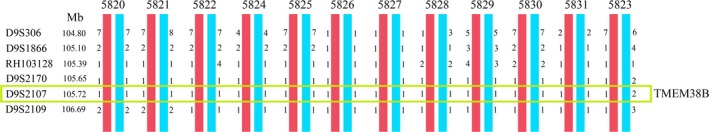
Haplotype analysis for the 12 probands with in an intragenic *TMEM38B* marker and five flanking markers. The green box indicates the intragenic marker D9S2107.

*TMEM38B* variants were identified in 12 probands and 9 affected family members (Fig. 1, 5820, 5821, 5826, 5827, 5828, and 5831). A previously reported homozygous exon 4 deletion (21 kb), g.32476_53457delinsATTAAGGTATA, p.(Gly152Alafs*5), was found in 11 probands. One proband was compound heterozygous for this 21 kb deletion and a nonsense variant, c.507G>A p.(Trp169*), located in exon 4. This latter variant is generally associated with a severe early‐onset form of OI characterized by bowing of the limbs and multiple fractures, mostly of the femur. In addition, some individuals have blue sclerae, bone deformities, and/or dentinogenesis imperfecta, but none have hearing loss (Table 1). We identified a shared haplotype between the 12 probands, indicating that the deletion most likely represents a founder alteration (Fig. 2).

### Alterations causing defects in collagen folding and cross‐linking

A novel homozygous small genomic deletion was identified in exon 3 of the *SERPINH1* gene, c.314_325del p.(Glu105_His108del). This in‐frame deletion caused the loss of four amino acid residues and resulted in a moderate to severe phenotype in an 18 months old proband presenting with blue sclerae, joint hypermobility, pectus deformity, osteopenia, and multiple recurrent fractures, in addition to general growth and developmental delay (Table 1).

Three different homozygous variants in the *FKBP10* gene were identified in four probands. The previously described nonsense variant c.1330C>T p.(Gln444*) segregated in four patients of the same family (Fig. 1, 5835). A novel splice site variant, c.391+4A>T, was identified in two probands originating from the same city, which indicates that the families could be related. The pedigree of the family containing eight patients harboring the splice site variant is presented in Figure 1_5833. mRNA studies revealed an out‐of‐frame skip of exon 2 of the *FKBP10* gene (data not shown). A third proband had a novel homozygous frameshift variant, c.1276del p.(Gln426Argfs*10) (Fig. 1, 5836). All affected individuals were diagnosed with Bruck syndrome based on recurrence of long bone fractures and congenital contractures typical of Bruck syndrome. In addition, scoliosis and/or pectus deformities (Table 1) occurred in accordance with previously published data (Alanay et al. 2010; Shaheen et al. 2011; Schwarze et al. 2013).

### Alterations causing defects in bone mineralization

In a proband aged 7 years, we identified a homozygous missense variant, c.242C>G p.(Ser81Cys), in exon 3 of the *SERPINF1* gene. The patient had recurrent and multiple fractures, but with normal sclerae and teeth (Table 1). The proband responded poorly to bisphosphonate treatment, as reported for patients with *SERPINF1*‐related OI (Homan et al. 2011; Venturi et al. 2012; Minillo et al. 2014).

## Discussion

We performed mutation analysis on a cohort of 49 Palestinian OI families. By using an OI gene panel NGS screening strategy, we identified variants of known OI genes in 41 probands, corresponding to a variant uptake rate of 84%. In contrast to the OI populations studied so far, more than half of the Palestinian OI probands (25/41) have a recessive form of OI due to high rates of consanguinity. Consanguinity was more prevalent in our study population than in the general Palestinian population (65% vs. 39%), indicating ascertainment bias in families with possible recessive genetic disorders (Table 1). Eight probands, of whom six belong to consanguineous families, did not have any disease‐causing variant in the known OI genes, reflecting further genetic heterogeneity in OI.

In total, we identified 28 different variants in nine OI genes, including 10 *COL1A1*, five *COL1A2*, three *BMP1*, three *FKBP10*, two *TMEM38B*, two *P3H1*, one *CRTAP*, one *SERPINF1*, and one *SERPINH1* (Table 1).

The phenotypes of patients with *COL1A1* and *COL1A2* variants (Table 1) were in line with previously reported genotype–phenotype correlations, with haploinsufficiency resulting in milder phenotypic abnormalities and pathogenic missense variants causing more severe phenotypes (Gentile et al. 2012; Vandersteen et al. 2014). However, the novel splice site variant c.3531+1G>T is associated with moderate phenotypic features, which is at odds with an earlier report that this variant resulted in mild OI (Willing et al. 1994).

We identified three novel homozygous *BMP1* missense variants affecting highly conserved amino acids located in the catalytic metalloprotease domain, which may thus interfere with the enzymatic activity of the BMP1/tolloid‐like protein. Hitherto, four *BMP1* missense variants have been reported (Asharani et al. 2012; Martinez‐Glez et al. 2012; Valencia et al. 2014; Cho et al. 2015; Syx et al. 2015). Two of them, c.747C>G p.(Phe249Leu) and c.808A>G p.(Met270Val), are in the same domain as the variants we identified (Fig. S1) and it diminishes BMP1/mTLD proteolytic activity, resulting in impaired secretion of the protein (Martinez‐Glez et al. 2012; Cho et al. 2015). The phenotypes of those two patients were severe in accordance with the phenotype of our patient harboring the c.818C>T p.(Ala273Val) variant. Nevertheless, the other two patients harboring the c.688C>G p.(Arg230Gly) and c.691G>T p.(Asp231Tyr) variants have a moderate and a mild phenotype, respectively (Table 1). Highly variable phenotypes associated with *BMP1* variants have been recently reported (Pollitt et al. 2016), but further investigation of the correlating molecular pathogenesis is needed. In agreement with previous observations (Baldridge et al. 2008), both variants of *CRTAP* and *P3H1* caused severe OI (Table 1). Notably, the *P3H1* splice site variant c.1080+1G>T has been previously described as the “West African allele’’ (Cabral et al. 2007; Pepin et al. 2013), but it seems to be more widely spread.

Remarkably, we found a recurrent exon 4 deletion p.(Gly152Alafs*5) in *TMEM38B* in 12 probands, making it the most frequent variant among the Palestinian OI patients. This variant has been reported previously in three Saudi Arabian families (Shaheen et al. 2012) and in three Israeli Arab Bedouin families (Volodarsky et al. 2013). The latter families have the same ancestry as the Palestinian population. Haplotype analysis suggests a founder effect for this particular variant. The genotype–phenotype correlation is in line with the previous reports, so we recommend evaluation of this variant in Palestinian families with moderate AR OI (Table 1).

The *SERPINH1* variant reported here is the first in‐frame genomic *SERPINH1* deletion to be identified, though its phenotype of moderately severe OI does not differ from the phenotype caused by variants generated by a premature termination codon (PTC) (Christiansen et al. 2010; Duran et al. 2015). Consistent with previous observations (Schwarze et al. 2013), patients with variants in the *FKBP10* gene were diagnosed with Bruck syndrome because of congenital contractures. Notably, OI severity varied widely (Table 1).

The proband harboring a *SERPINF1* missense variant has a milder phenotype than the patients previously reported (Becker et al. 2011; Homan et al. 2011; Caparros‐Martin et al. 2013; Cho et al. 2013; Minillo et al. 2014; Stephen et al. 2015), possibly because the mutant PEDF protein retains some activity. Although the c.242C>G p.(Ser81Cys) missense variant has been reported as a variant with uncertain clinical significance in the Clinvar database (Variation ID 218613) and with an allele frequency in ExAC of about 0.1%, the amino acid is well conserved and is located directly next to a putative receptor binding site of the PEDF protein (Fig. S2). Missense variants in *SERPINF1* were found to underlie otosclerosis in three families (Ziff et al. 2016), but our patient aged 7 years had no hearing impairment.

In conclusion, AR forms of OI are the most prevalent (>60%) in the Palestinian OI population. This finding emphasizes the importance of genetic analysis of AR OI genes in Palestinians with OI in order to reduce the risk of this devastating disorder. A *TMEMB38B* deletion was found to be the most common variant among the Palestinian OI patients. In eight probands we did not identify any disease‐causing variant in the known OI genes, making them suitable for exome sequencing in order to identify the underlying genetic defects. Such analysis will probably lead to the identification of new OI genes.

## Conflict of Interest

The authors declare no conflict of interest.

## Supporting information


**Figure S1.** *BMP1* catalytic metalloprotease domain.
**Figure S2.** PEDF homologs and protein structure.
**Table S1.** Linkage analysis markers.
