# Supplementary material for: Genetic analysis of osteogenesis imperfecta in the Palestinian population: molecular screening of 49 affected families
Source: Mol Genet Genomic Med. 2017 Nov 18;6(1):15–26. doi: 10.1002/mgg3.331 (PMC5823677; doi:10.1002/mgg3.331)
Supplement: Supplementary file 1 — Figure S1. BMP1 catalytic metalloprotease domain. Figure S2. PEDF homologs and protein structure. Table S1. Linkage analysis markers. [file MGG3-6-15-s001.docx]

**Supplementary Table S1: Linkage analysis markers**

| **Marker** | **Primer 1** | **Primer 2** | **Product size** |  | **Product size identified in probands** | | | | | | | |
| --- | --- | --- | --- | --- | --- | --- | --- | --- | --- | --- | --- | --- |
|  |  |  |  | **1** | | **2** | **3** | **4** | **5** | **6** | **7** | **8** |
| D9S306 | AGCTCATCCTGGCTTTAAAC | GGTGGAATATGTTTTTATTAGC | 122-149 | 122 | | 125 | 130 | 132 | 136 | 140 | 142 | 149 |
| D9S1866 | CGGCCTGGTACTCAATGTAT | GCCCTCACATCCATCCTATC | 276-289 | 280 | | 285 | 287 | 289 |  |  |  |  |
| RH103128 | GAGCAATGATCTCATTTGCA | AAGTAAACATGTATAGGAAAAAGCT | 277-292 | 277 | | 288 | 292 | 296 |  |  |  |  |
| D9S2170 | CCAGCCTGGTCAACATAGTG | GTAGATGGGGTAGGGAAGAG | 217-225 | 217 | | 225 |  |  |  |  |  |  |
| D9S2107 | GGTGGGTCAGTTTCAAAAGC | TCACTGGAAAGCAGAGCAGA | 208-223 | 217 | |  |  |  |  |  |  |  |
| D9S2109 | CAAATTCTCATAACTTCCATGC | TATCTCTTTTGGTTACCTAATTCC | 234-238 | 234 | | 236 |  |  |  |  |  |  |

**Figure S1**: *BMP1* catalytic metalloprotease domain.


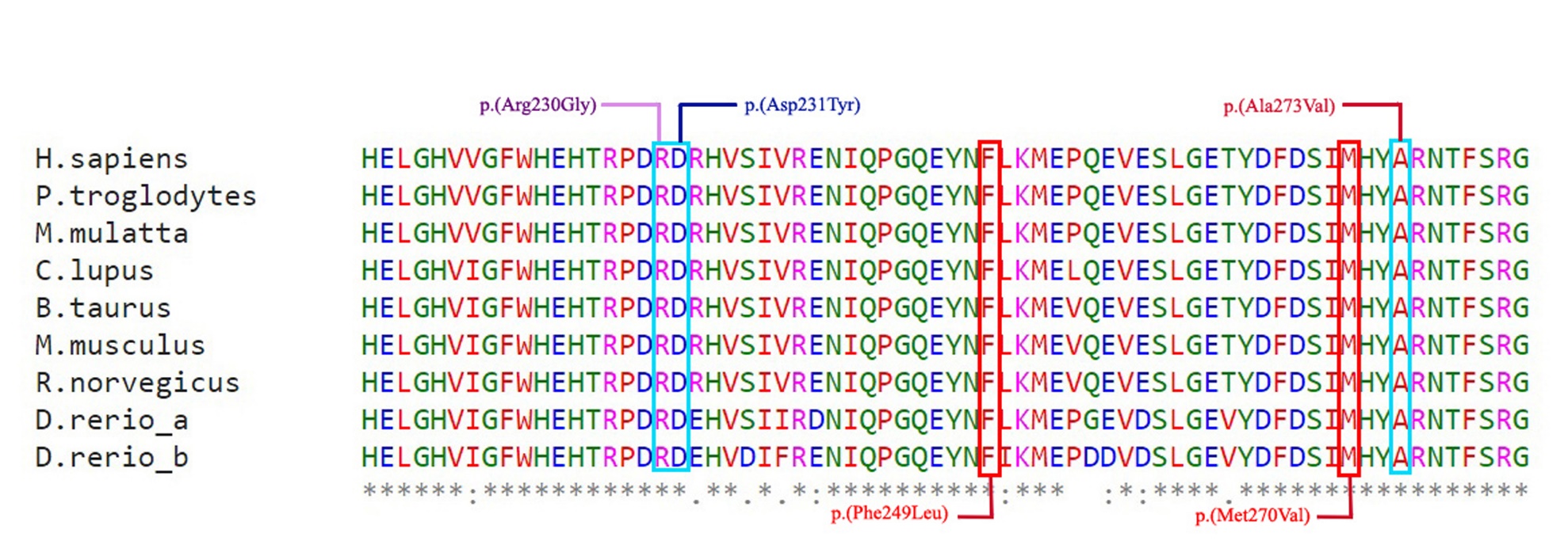


Alignment of the amino acid sequence in the highly conserved catalytic metalloprotease domain of *BMP1*. The mutated residues p.(Arg230), p.(Asp231) and p.(Ala273), are indicated above and the previously reported missense mutations p.(Phe249) and p.(Met 270) are indicated below. Nomenclature refers to NCBI RefSeq *BMP1,* NM_006371.4 / NP_006362.1

**Figure S2:** PEDF homologs and protein structure.

A


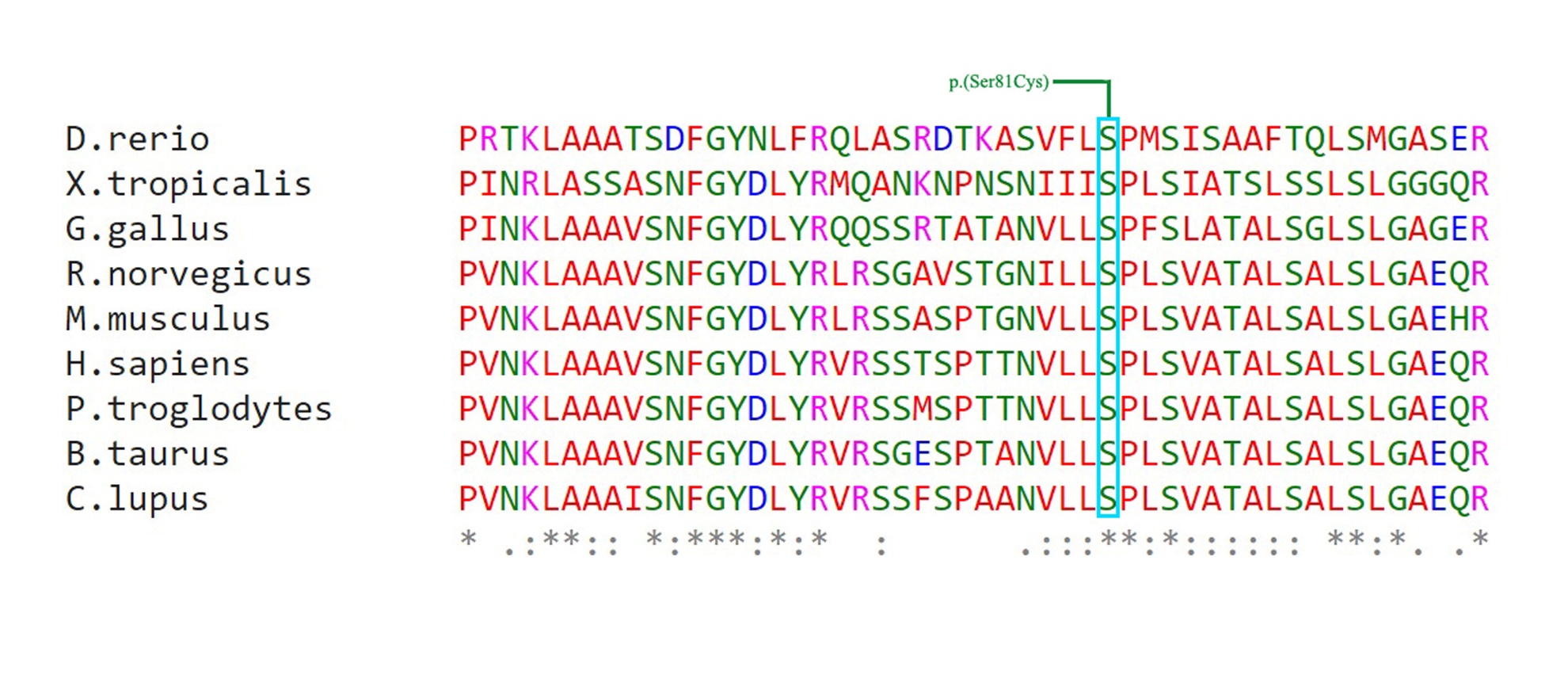


A) Amino acid alignment of PEDF homologs; the mutated residue p.(Ser81) is indicated. Nomenclature refers to NCBI RefSeq *SERPINF1*, NM_002615.5 / NP_002606.3


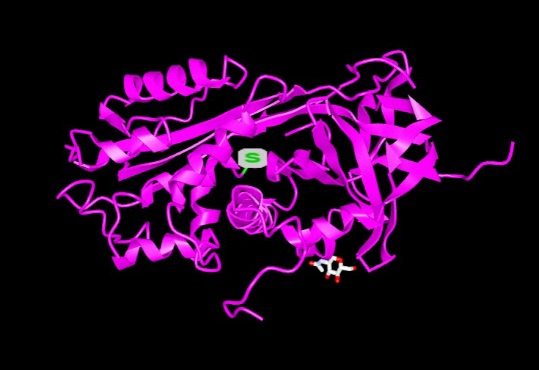
B

B) PEDF 3D protein structure^1^ indicating the central location of the amino acid serine, which is located directly adjacent to a putative receptor binding site of the PEDF protein.

^1^ The protein structure was published by Simonovic et al., 2001, and the Cn3D software was used for visualization.
